# Intimate partner violence and HIV testing during antenatal care: A latent class analysis to identify risk factors for HIV infection in mothers and their children in the United Republic of Tanzania

**DOI:** 10.1371/journal.pgph.0000831

**Published:** 2022-08-12

**Authors:** Francisco A. Montiel Ishino, Claire Rowan, Joel Seme Ambikile, Donaldson F. Conserve, Diana Lopez, Melanie Sabado-Liwag, Faustine Williams

**Affiliations:** 1 Division of Intramural Research, National Institute on Minority Health and Health Disparities, National Institutes of Health, Bethesda, Maryland, United States of America; 2 Transdisciplinary Center for Health Equity Research, College of Education and Human Development, Texas A&M University, College Station, Texas, United States of America; 3 School of Nursing, Muhimbili University of Health and Allied Sciences, Dar es Salaam, United Republic of Tanzania; 4 Prevention and Community Health, Milken Institute School of Public Health, The George Washington University, Washington, District of Columbia, United States of America; 5 Department of Public Health, California State University, Los Angeles, Los Angeles, California, United States of America; Conservatoire national des arts et metiers, FRANCE

## Abstract

Intimate partner violence has adverse effects on mother’s overall health and prevention of mother to child HIV transmission. To identify and examine subgroups of mothers experiencing intimate partner violence and the likelihood of HIV testing during antenatal care, we conducted a latent class analysis using data from the Tanzania Demographic and Health Survey 2010 (N = 2,809). Intimate partner violence included mother’s experiences with partners’ controlling behaviors, as well as emotional, physical, and sexual violence. The outcome was mother’s accepting HIV testing offered during their antenatal care visit. Covariates included mother’s level of education, rural/urban residence, and prevention of mother to child HIV transmission talk during antenatal care visit. The latent class analysis indicated a three-class solution was the best model and identified the following profiles: mothers with no experience of intimate partner violence (61% of sample) with a 90.5% likelihood of HIV testing; mothers with moderate levels of intimate partner violence (26%) with an 84.7% likelihood of testing; and mothers with extreme levels of intimate partner violence (13%) with an 82% likelihood of testing. An auxiliary multinomial logistic regression with selected covariates was conducted to further differentiate IPV profiles, where mothers with extreme levels of intimate partner violence had 57% increased odds [95%CI:1.06–2.33, p = .023] of living in rural areas compared to mothers with no experience of intimate partner violence. Our person-centered methodological approach provided a novel model to understand the impact of multiple intimate partner violence risk factors on antenatal care HIV testing to identify mothers in need of interventions and their children at highest for parent to child HIV transmission. Our model allows person-centered interventional designs tailored for the most at-risk subgroups within a population.

## Introduction

In 2002, an estimated 1.5% of children from the United Republic of Tanzania (Tanzania) under the age of 5 were living with HIV, representing a prevalent health burden in the East African nation [1]. Estimates from 2020 approximated that 110,000 children aged 0 to14 were newly infected, with a projected antiretroviral therapy (ART) coverage of 48% [2]. Many children are exposed to the retrovirus through their mother living with HIV, and are left untreated due to lack of testing while pregnant or during antenatal care (ANC) [3]. There have been major public health efforts since 2010 to treat people living with HIV with ART and to prevent mother-to-child transmission (PMTCT) in Tanzania [4]. One major factor often found missing from public health efforts is the maternal environment and experiences therein.

Current research has revealed potential targets for HIV care access than can also be used in the context of PMTCT. For example, there is an overwhelming significance of proximity to testing facilities along with stigma associated with HIV testing that limits a patient’s initial access to and continuity of HIV care [5]. Individuals who are diagnosed as HIV positive need to receive care to protect themselves from future opportunistic diseases and secondary HIV infections in the community [6]. Yet, when discussing access to HIV care, a vulnerable group that must be focused on are survivors of intimate partner violence (IPV) [7]. Previous studies link IPV to HIV infection due to forced sexual intercourse, lack of disclosing HIV status to a partner, or a limited ability to negotiate preventative behaviors [8]. Previous research points to high prevalence of IPV in Tanzania, with lifetime IPV exposure at 65% among ever-married or ever-partnered women [9].

IPV exposure can have severe consequences on both maternal and child health, with 38.8% of pregnant women reporting having been exposed to at least one type of violence in a recent study [10]. IPV during pregnancy has been linked to adverse health outcomes such as low-birth weight and pre-term delivery, frequency of sexually transmitted infections, PTSD, depression, alcohol use, and injury [10–14]. The pervasiveness of male intimidation traditionally found within this community is hypothesized to influence female decision-making regarding HIV testing for themselves and their children. Sexual and physical violence experience increases negative health outcomes and risk taking. These outcomes are then perpetuated with fathers, mothers, and their children, which leads to a perpetual cycle of possible HIV continuation/transmission to children even in light of interventions that exist to PMTCT [15].

Novel targets for intervention and prevention of HIV in children under 5 may lie within the interpersonal domain of the mother. For example, education level has been shown to lead to lower testing rates as higher education has been linked to better knowledge of the consequences of HIV infection [16]. Previous studies, however, show that women with lower levels of education are twice as likely to experience IPV than women with more education [17]. This relationship indicates that a woman’s lack of education could influence not only her experience of violence but affect her ability to be tested for HIV. Our study examined the latent or unobserved interactions from the IPV domains of the mother to determine possible points of prevention and intervention.

Therefore, to correctly determine the influences on HIV testing with respect to the interpersonal domain of the mother, we reached outside the literature, examining nationally representative data from the 2010 Demographic Health Survey (DHS) for Tanzania. Our research focus was to identify mothers’ profiles on likelihood of HIV testing during ANC based on reports of IPV while assessing the relationship of critical environmental determinants on identified profiles.

## Materials and methods

We examined unobservable–or latent–subgroups of Tanzanian mothers experiencing IPV and their likelihood of taking an HIV test during their ANC visit. We used latent class analysis (LCA) on the 2010 DHS for Tanzania as it provided ANC, HIV testing, and IPV data through surveys and interviews from individual mothers. The DHS survey is deployed by the DHS Program, which has provided technical assistance since 1984 on more than 300 surveys to more than 90 countries. Health information on individual pregnant women was collected from interviews and surveys in Tanzanian households, ranging between 3,000 to 9,000 [18]. Households were selected by the DHS through a stratified random sample to be representative of urban and rural areas. For further detail see 2010 TDHS [18]. Pregnant women self-reporting any type or level of IPV numbered 4,633. For the purposes of our study, we assessed pregnant mothers that were offered HIV testing during an ANC visit with any self-report of IPV (N = 2,809). The data analyzed for this study are restricted, and were requested from The DHS Program (https://dhsprogram/com/data/new-user-registration.cfm). Data are available upon request from The DHS Program. The Texas A&M University Institutional Review Board (IRB) determined that our protocol did not involve human subjects and was excluded from IRB review.

### Latent construct

The distal outcome of PMTCT was based on mother’s HIV testing during ANC visit (no/yes). The latent construct of IPV was measured using the following indicators from mother’s experiences with most recent husband or partner during the last 12 months: (a) the number of controlling behavior events reported (categorized as 0; 1; 2; 3 or more); (b) emotional violence (no/yes); (c) physical violence, both less severe and severe (no/yes); and (d) sexual violence (no/yes). The observed indicator of controlling behaviors was based on counts from the following experiences that included: (1) partner became jealous or angry if [mother] talked to other men; (2) frequently accused [mother] of being unfaithful; (3) does not permit [mother] to meet her female friends; (4) tries to limit [mother’s] contact with her family; (5) insists on knowing where [mother] is at all times; and/or (6) does not trust [mother] with money. The observed indicators of emotional violence was based on responses from mothers’ that answered: (1) “yes” to ever experiencing one or more of the following (a) “said or did something to humiliate her in front of others”; (b) “threatened to hurt or harm her or someone she cared about”; and/or (c) “insulted her or made her feel bad about herself”; and reported any or all of these events as occurring (2) “often” or “sometimes”. Physical violence was categorized as less severe and severe by the DHS using the following criteria: (1) less severe violence was operationalized (a) “pushing, shaking, and throwing something at female partner”; (b) “slapping”; (c) “twisting arm or pulling hair”; and/or (d) “punching with partner’s fist or with something that could hurt her”; and (2) severe violence was operationalized as (a) “kicking, dragging, or beating up”; (b) “choking or burning”; and/or (c) “threatening or attacking with some type of weapon”. The observed indicator of sexual violence was based on responses from mothers’ that answered (1) “yes” to ever experiencing one or more of the following (a) “physically forced her to have sexual intercourse with him when she did not want to; (b) “physically forced her to perform any other sexual acts she did not want to”; and/or (c) “forced her with threats or in any other way to perform sexual acts she did not want to”; and reported any or all events as occurring (2) “often” or “sometimes”.

*Covariates* were used in an auxiliary multinomial logistic regression to assess latent class membership of identified profiles. Covariates from environmental determinants included were mothers’ level of education (below or at/above primary elementary level), household residence designations (rural/urban), and if the mother discussed PMTCT with medical or health staff during ANC visits (no/yes).

### Latent class analysis

To select the best fitting LCA model, we employed a comparative approach that compared multiple models (i.e., 1-class solutions and onward) by Bayesian information criterion (BIC), sample size adjusted (ssa) BIC, Lo-Mendell-Rubin (LMR) adjusted likelihood-ratio-test (LRT), and entropy [19]. Entropy provides an index of reliability for separation of classes. The model favored will have the most parsimonious solution with lowest BIC and ssaBIC [19]. LMR LRT is used to facilitate the selection process through comparison of the *k*-1 and *k* class solutions, where a *p* > .05 would mean that while both models explain the observations equally the *k*-1 should be selected in favor of parsimony [19]. All analyses were carried out on Mplus 8.4 (Muthén & Muthén). Analyses were weighted, with strata and clusters used to account for the complex survey design of the 2010 Tanzania DHS and survey non-response. An auxiliary multinomial logistic regression was then performed using covariates to further establish class membership. All analytical files are available upon reasonable request.

## Results

As reported in Table 1, 73% of mothers offered HIV testing during their ANC were tested. We found that within the past 12 months, the majority of mother’s reported experiencing high levels of controlling behaviors (i.e., 3 or more controlling behaviors at 36.2%), less severe violence (40.2%), severe violence (15.2%), and sexual violence (15.4%). A majority of mothers also reported in the past 12 months not having experienced emotional violence (61.9%). Mothers also reported having below a primary level of education (81.1%), residing in rural designated area (71.6%), and receiving a PMTCT talk during ANC (71.4%).

**Table 1:** Sample descriptives (N = 4,633).

	N	%
Number of controlling behaviors (n = 4,447)		
0	982	22.1
1	971	21.8
2	886	19.9
3+	1,608	36.2
Experienced any emotional violence (n = 4,446)		
No	2,754	61.9
Yes	1,692	38.1
Experienced any less severe violence (n = 4,447)		
No	2,661	59.8
Yes	1,786	40.2
Experienced any severe violence (n = 4,431)		
No	3,759	84.8
Yes	672	15.2
Experienced any sexual violence (n = 4,447)		
No	3,761	84.6
Yes	686	15.4
HIV testing during ANC visit (n = 2,635)		
No	711	27.0
Yes	1,924	73.0
Primary level education		
Below	3,757	81.1
At or above	876	18.9
Household residence designation		
Urban	1,316	28.4
Rural	3,317	71.6
PMTCT talk during ANC (n = 3,294)		
No	942	28.6
Yes	2,352	71.4

### Latent class analysis

**Fig 1:**
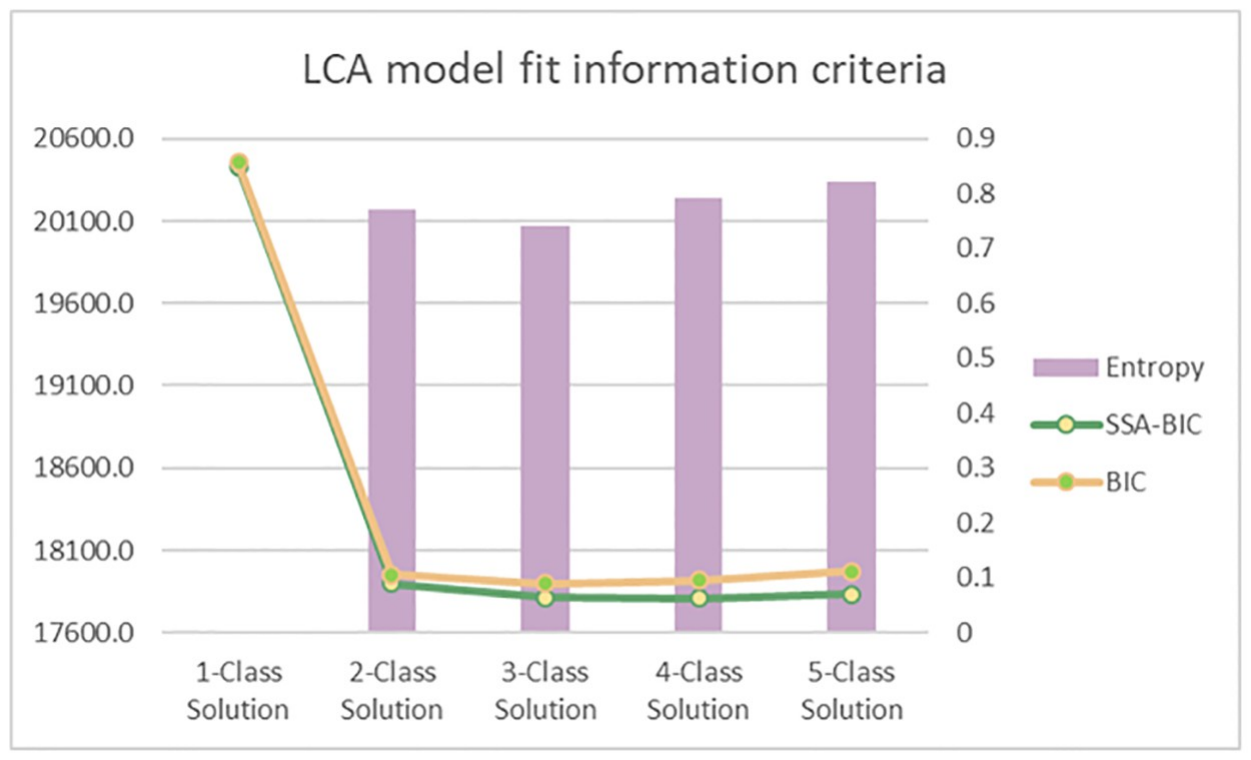
Latent class analysis information criterion for the selection of best n-class solution for interpretation.

The 3-class solution was selected based on the overall lowest BIC and the non-significant LMR LRT *p*-value of the 4-class solution, which indicated that the *k*-1class solution or 3-class solution is favored (Table 2). The profiles for the 3-class solution were named using the conditional probabilities of observed indicators of IPV within and between classes. See Fig 1 for more detail.

**Table 2:** Latent class analysis model fit assessment.

	BIC	ssaBIC	Entropy	LMR LRT	*p*
1-Class Solution	20,456.6	20,431.1	-	-	-
2-Class Solution	17,954.3	17,900.3	0.77	2538.2	0.000
3-Class Solution	17,897.4	17,814.8	0.74	126.6	0.022
4-Class Solution	17,918.7	17,807.5	0.79	49.5	0.126
5-Class Solution	17,972.9	17,833.1	0.82	17.0	0.574

Notes. BIC = Bayesian information criterion;

ssaBIC = sample size adjusted Bayesian information criterion;

LMR LRT = Lo-Mendell-Rubin adjusted likelihood-ratio-test

**Class 1**, or mothers with little to *no* experience of IPV (61% of sample) were 90.5% likely to take an HIV test during their ANC visit. Mothers in Class 1 had the highest conditional probabilities of having experienced no controlled IPV events from their most recent husband or partner (35.3%), and reported no experience with emotional violence (96.1%), or sexual violence (98.7%). They also had the highest probabilities of not experiencing any violence, whether less severe or severe (92.8% and 99.9%, respectively).

**Class 2**, or mothers with *moderate* experience of IPV (26%) of the sample) were 84.7% likely to take an HIV test during their ANC visit. This class grouping had high probabilities of experiencing more than three controlled IPV events (52.5%), emotional violence (71.8%), less severe violence (66.8%), and sexual violence (27.1%) with their most recent husband or partner. Mothers in Class 2 reported no experience with severe violence (100%).

Mothers with *extreme* experiences of IPV, **Class 3**, (13% of the sample) had the lowest likelihood (82.0%) to take a test for HIV during their ANC visit. Mothers in Class 3 had the highest conditional probabilities of experiencing more than three controlled IPV events (65.5%), emotional violence (91.2%), less severe (96.9%) and severe violence (90.3%), and sexual violence (44.0%). See Table 3 for more details on the latent classes.

**Table 3:** Latent classes of intimate partner violence (N = 2,809).

	Class 1	Class 2	Class 3
	1,715	718	376
	61%	26%	13%
Number of control issues			
0	0.353	0.113	0.065
1	0.282	0.167	0.110
2	0.187	0.195	0.170
3+	0.177	0.525	0.655
Experienced any emotional violence			
No	0.961	0.282	0.088
Yes	0.039	0.718	0.912
Experienced any less severe violence			
No	0.928	0.332	0.031
Yes	0.072	0.668	0.969
Experienced any severe violence			
No	0.999	1.000	0.097
Yes	0.001	0.000	0.903
Experienced any sexual violence			
No	0.987	0.729	0.560
Yes	0.013	0.271	0.440
HIV testing during ANC visit			
No	0.095	0.153	0.180
Yes	0.905	0.847	0.820

### Auxiliary multinomial logistic regression of covariates

As seen in Table 4, the auxiliary multinomial logistic regression analysis found that mothers with extreme experiences of IPV (Class 3) were 57% more likely (95% CI = 1.06–2.33) to reside in a rural area when compared to mothers with little to no experience of IPV (Class 1).

**Table 4:** Auxiliary multinomial logistic regression using Class 1 or mothers with little to no experience of IPV as reference (n = 2,797).

	Class 2				Class 3			
		95% CI				95% CI		
	OR	Lower	Upper	*p*	OR	Lower	Upper	*p*
Mother with elementary level education or above	1.11	0.70	1.76	0.65	0.93	0.61	1.42	0.74
Rural residence	1.21	0.79	1.84	0.38	**1.57**	**1.06**	**2.33**	**0.02**
Antenatal visit PMTC talk	0.89	0.57	1.39	0.60	0.84	0.56	1.27	0.41

## Discussion

While IPV has been linked to chronic burdens of disease and negative health outcomes in mothers and their child(ren), our study is among the first to link IPV experiences to the likelihood of HIV testing during pregnancy. Our study built upon the call to understand the negatively synergistic effects of IPV that affect pregnant women; to not only address the risks of violence and HIV infection to them as mothers but the subsequent long-term effects to their children [20]. As such, we identified three mutually exclusive profiles of IPV (i.e., little to no IPV, moderate IPV, and extreme IPV) and the respective likelihoods of HIV testing during ANC. Overall, we found that the more IPV mothers experienced, the likelihood of ANC HIV testing decreased. We also found that mother’s level of education and talks concerning HIV transmission from mother-to-child during their ANC visit were not significant factors in differentiating IPV profiles and their likelihood of HIV testing during their ANC visit.

The profile of mothers with little to no experience of IPV had the lowest likelihoods of IPV events (emotional, less severe and severe violence, and sexual) while having the highest likelihood of HIV testing during their ANC visit compared to all profiles. Mothers in the extreme IPV profile had the highest likelihoods of experiencing emotional violence, severe physical violence, and sexual violence, as well as more controlling behaviors while having the lowest likelihood to be tested for HIV during their ANC visit compared to all profiles. Geographic residence, however, was found to be a differentiating factor between the profiles of lowest and highest IPV. Mothers living in rural Tanzania were found to have 57% increased odds of being in the extreme IPV profile when compared to mothers in the little to no IPV profile.

Intervention programs for IPV in rural Tanzania exist [21], however, lack of resources and access, as well as cultural norms and behaviors may affect the efficiency and efficacy of programs. Education has often been considered a critical factor in mitigating IPV, yet Tanzania has compulsory education up to the primary level [22]. In our study sample we found, however, that less than 19% were at or below a primary level education. We hypothesize that this may explain why education was not found to be a significant differentiating factor between IPV profiles. Moreover, this may also suggest as to why PMTCT talks during ANC visits may have also not been significant differentiating factors between profile. We cannot speak further as to the possible shortcomings or strengths of the PMTCT talks were as we do not have the information available. Regardless, we must acknowledge that there are various points of intervention that can be introduced given our findings for Tanzania.

The first would be to continue compulsory primary level education, and push for enrollment into secondary level [23]. We acknowledge that there are several barriers such as enforcement in rural areas, as well as structural issues like socioeconomic status and family dynamics. Through education can we then help create gender and economic equality, as well as empower women [24, 25]. Empowerment, however, is culture specific. Efforts must be taken to advance women at the community level to identify and address detrimental behaviors from their partners to begin normative change that are culturally empathic and appropriate for Tanzania. Community-based education would also include tailored discussions on empowering spousal communication, gender-based violence, and HIV stigma that hinders access to testing. By creating a culturally empathic models that promotes normative change we can help not only address IPV but improve HIV testing during ANC. Cultural dynamics and familial infrastructure also play significant roles on the success of evidence-based implementation strategies like community savings groups, lifelong ART, or male condom education. For example, community savings groups provide female sex workers with the ability to collectively control some of the risks and vulnerabilities that they find in their jobs [26, 27].

Pregnant women who experience IPV may also be victim to increased barriers. Barriers include limited access to ANC and navigation towards services like HIV testing and treatment. Furthermore, treatment for mothers living with HIV may result in poor adherence to ART medication. This may also affect pre- and post-exposure prophylactic treatment, as well as decrease exposure to primary care prevention interventions like PMTCT that would decrease mother to child transmission (MTCT) of HIV during pregnancy, labor/delivery, or during breastfeeding [28–30]. Testing and knowing HIV status is a major determinant to ANC behavior, which in turn, is pertinent to MTCT outcomes. Increased IPV, social stigma, and long-term abandonment are negative consequences to HIV testing and positive results during pregnancy, especially if the couple is serodiscordant [31].

Our study has multiple strengths, such as the use of consistent variables from other studies for comparison and corroboration [11] in our person-centered model. We included multiple interacting IPV experiences (psychological, physical, and sexual violence) in our model that would be difficult in variable-centered modeling. Our analysis is among the first to use a person-centered approach to identify profiles of HIV risk for MTCT using pregnant mother’s experiences of IPV. Moreover, our approach not only reveals the syndemic vulnerability of mothers and their children but allows for rapid risk assessment and intervention strategies. Our study, nonetheless, is not without limitations. The large cross-section survey design precludes our ability to determine any temporal relationships and causality among any of the risk and protective factors, and HIV test results could not be ascertained nor used in our analyses. While we had these limitations, we found that there are nuanced profiles in which interventions in ANC did not associate with outcome of testing. Future direction will include multiple time points to identify causal and transitional factors salient to HIV testing among pregnant mothers.

## Conclusion

In summary, our person-centered approach revealed a syndemic vulnerability among pregnant mothers and their experiences with IPV to intervene on mother to child HIV transmission. We identify risk profiles among pregnant mothers to decrease possible HIV transmission to infants and overall promote PMTCT programs. In order to address IPV among pregnant mothers in Tanzania, many strategies can be used including empowering women to address IPV, lifelong ART, male condom education, and intervention at breastfeeding. With regards to IPV experience, those in the highest IPV risk class had the lowest chance of HIV testing in an ANC setting. Our study provides a model to help identify groups at highest risk and rapidly deploy intervention programs to help in the tailoring and evaluate of subsequent interventions and prevention programs. This allows for identification of subgroups to move from umbrella interventions to tailored interventions, increasing adoption and adherence by accounting mother’s experiences while promoting their wellbeing and that of their child.
